# Associations of timing of food intake with energy intake, eating behaviour traits and psychosocial factors in adults with overweight and obesity

**DOI:** 10.3389/fnut.2023.1155971

**Published:** 2023-05-30

**Authors:** Raphaëlle Jacob, Angelo Tremblay, Véronique Provencher, Shirin Panahi, Marie-Ève Mathieu, Vicky Drapeau

**Affiliations:** ^1^School of Nutrition, Université Laval, Quebec, QC, Canada; ^2^Centre Nutrition, santé et société (NUTRISS), Institute of Nutrition and Functional Foods (INAF), Université Laval, Quebec, QC, Canada; ^3^Quebec Heart and Lung Institute Research Centre, Université Laval, Quebec, QC, Canada; ^4^Department of Kinesiology, Faculty of Medicine, Université Laval, Quebec, QC, Canada; ^5^Department of Physical Education, Faculty of Education, Université Laval, Quebec, QC, Canada; ^6^School of Kinesiology and Physical Activity Sciences, Faculty of Medicine, Université de Montréal, Quebec, QC, Canada; ^7^Sainte-Justine University Health Centre Research Centre, Université de Montreal, Quebec, QC, Canada

**Keywords:** timing of food intake, obesity, eating behaviours, late eating, psychosocial factors, energy intake

## Abstract

**Introduction:**

Whether a late distribution of food intake impacts obesity through increased energy intake remains uncertain and the behavioural characterization of late eating needs to be further investigated. The first objective of this study was to assess the associations between late eating and body mass index (BMI) and total energy intake (TEI), and whether TEI mediates the association between late eating and BMI. The second objective was to assess the associations between late eating and eating behaviour traits or psychosocial factors and whether eating behaviour traits mediate the association between late eating and TEI.

**Methods:**

Baseline data from 301 individuals (56% women, age = 38.7 ± 8.5 years; BMI = 33.2 ± 3.4 kg/m^2^), who participated in four weight loss studies were used in this cross-sectional study. Total energy intake was assessed using a three-day food record from which the percentage of TEI after 17:00 and after 20:00 was calculated. Eating behaviour traits and psychosocial factors were assessed with questionnaires. Pearson correlations and mediation analyses adjusted for age, sex, underreporting of energy intake, sleep duration and bedtime were performed.

**Results:**

Percent TEI after 17:00 and after 20:00 were associated with TEI (*r* = 0.13, *p* = 0.03 for both), and TEI mediated the association between percent TEI after 17:00 and BMI (*β* = 0.01 ± 0.01, 95% CI: 0.001, 0.02). Percent TEI after 17:00 was associated with disinhibition (*r* = 0.13, *p* = 0.03) and percent TEI after 20:00 was associated with susceptibility to hunger (*r* = 0.13, *p* = 0.03), stress (*r* = 0.24, *p* = 0.002) and anxiety (*r* = 0.28, *p* = 0.0004). In women, disinhibition mediated the association between percent TEI after 17:00 and TEI (*β* = 3.41 ± 1.43, 95% CI: 0.92, 6.47). Susceptibility to hunger mediated the association between percent TEI after 20:00 and TEI (*β* = 0.96 ± 0.59, 95% CI: 0.02, 2.34) in men and women.

**Conclusion:**

Late eating is associated with TEI and suboptimal eating behaviours which could contribute to explaining the association between timing of food intake and obesity.

## 1. Introduction

Recent evidence indicates that the timing of food intake is a risk factor for obesity and associated comorbidities such as type 2 diabetes and cardiovascular diseases (1–3). Cross-sectional, prospective and interventional studies have shown that eating later during the day was associated with obesity or greater adiposity (4–9), weight gain (10) and reduced weight loss (11–16).

The distribution of food intake throughout the day is an important synchronizer of peripheral clocks, located in many organs and tissues (1). Consuming a high proportion of energy later during the day and into the night can produce chronodisruption, a state where peripheral clocks are out of synchrony with the central clock, located in the suprachiasmatic nucleus and aimed to synchronise behaviours with environmental cues (1, 2). A state of chronodisruption can impact many physiological processes (1, 2). However, the mechanisms explaining the increased susceptibility to obesity among late eaters remain to be fully understood. Some studies, but not all (17–20), have shown no associations between late eating and total energy intake (TEI) either cross-sectionally (13) or during weight loss (12, 14, 21). Based on these results and those of experimental studies exploring the effect of timing of food intake on metabolism (1, 22–24), it has been suggested that late eating impacts body weight mainly through energy expenditure (e.g., lower thermic effect of foods) (24) rather than through energy intake (1). However, late eating resulted in higher daily appetite sensations in two recent cross-over randomised controlled trials in which food intake was fully controlled (25, 26). These results seem inconsistent with the previous hypothesis and suggest that energy intake may be involved in the association between timing of food intake and obesity, but this needs to be assessed in well-designed studies conducted under free-living conditions.

In studies where food intake is not fully controlled, the lack of influence of late eating on energy intake remains uncertain as most studies relied on self-reported dietary assessment tools without consideration of misreporting of energy intake which can result in attenuated or misleading associations (27). Accounting for misreporting of energy intake may be particularly relevant in this context as underreporting is associated with obesity and is more likely to occur with foods of low nutritional value that may be perceived as socially undesirable (28) and that are associated with evening preferences (29, 30). Moreover, late eaters have been characterized as being more prone to eating when stressed, overeating at night and eating while watching television (13). Disinhibition, which refers to an overconsumption of food triggered by different cues (31), habitual and emotional susceptibility to disinhibition, and binge eating severity have also been associated with a higher proportion of TEI consumed as evening snacks (32). Eating behaviour traits such as disinhibition, emotional eating and binge eating have been associated with higher energy intake, weight gain and obesity (33–40). Although the behavioural characterization of late eaters in the literature is scarce, these results support the hypothesis that late eating may also impact body weight through energy intake.

To improve obesity treatment and prevention, there is a need to better understand how late eating impacts body weight. Importantly, more studies accounting for systematic bias associated with dietary assessment tools or using objective measurement of dietary intake are needed to shed light on the possible association between late eating and energy intake. Expanding the behavioural characterization of late eaters is also important as it could help develop targeted interventions for these individuals. To our knowledge, only two studies have assessed eating behaviour traits associated with late eating (13, 32). Based on results from these two previous studies showing that late eating is associated with eating in response to negative emotions (13, 32), it is possible that late eaters are characterized by higher levels of psychosocial factors such as stress, anxiety and depressive symptoms, but this remains to be assessed.

The first objective of this study was to assess the associations between a late distribution of food intake (i.e., late eating), body mass index (BMI) and TEI and to determine whether TEI mediates the association between late eating and BMI, while considering underreporting of energy intake. The second objective was to examine the associations between late eating, eating behaviour traits and psychosocial factors (i.e., stress, anxiety and depressive symptoms), and to investigate whether eating behaviour traits related to overeating (e.g., disinhibition and susceptibility to hunger) mediate the association between late eating and TEI. We hypothesised that late eating is associated with TEI and BMI and that TEI mediates the association between late eating and BMI. We also hypothesised that late eating is associated with overeating-related eating behaviour traits and that these traits mediate the association between late eating and TEI.

## 2. Materials and methods

### 2.1. Participants

This cross-sectional study included baseline data from 301 individuals with overweight or obesity from the Weight Loss Intervention Studies (WeLIS) Cohort, which includes four previous weight loss studies with similar designs conducted at Université Laval (41–44). These studies aimed to assess the effect of various supplements (i.e., probiotic, calcium and vitamin D, or a multivitamin and mineral) compared to placebo as part of a 12–15-week energy-restricted intervention (42–44), or the effect of a non-restrictive satiating diet compared to standard nutritional guidelines (i.e., Canada’s Food Guide 2007) for 16 weeks (41), on weight loss. Inclusion criteria were to be aged 20 to 55 years, living with overweight or obesity, having a body weight variation of less than 4 kg for at least 2 months before the study, being inactive to low active (i.e., maximum of three periods of 30 min of low to vigorous intensity physical activity per week), being in apparent good health (i.e., absence of any disease or condition that may pose a risk to the participant or alter the results of the study) based on a standard medical examination performed by a physician during the screening visit of each study, having no comorbidities such as type 2 diabetes or cardiovascular diseases, not taking medications or supplements that could impact study outcomes, consumption of less than 10 alcoholic beverages per week and a maximum of 2 drinks per day, consumption of less than 5 cups of coffee per day, not being pregnant or lactating and absence of menopause for women. Individuals working at night during the completion of the three-day food record (*n* = 1) and those who completed less than 2 days of food record (*n* = 3) were excluded from the present study (Supplementary Figure S1). Each study was approved by the Research Ethics Board of Université Laval and written informed consent was obtained from each participant before the study.

### 2.2. Anthropometric measurements

Anthropometric measurements were performed according to standardised procedures recommended at the Airlie Conference (45). Body weight was measured to the nearest 0.1 kg using a digital scale and height was measured to the nearest 0.1 cm using a standard statiometer. Body mass index was calculated as kg/m^2^.

### 2.3. Dietary assessment and distribution of food intake

Dietary intakes were assessed with a three-day food record completed on two weekdays and one weekend day at baseline (46). Participants received instructions on how to complete the food record and measure quantity of food consumed. The research dietitian reviewed the completed food record with the participant to ensure that all information was clear and complete. Food records were analyzed using the Nutrific software (47) linked to the Canadian Nutrient File version 1997 or 2005, depending on the studies (48, 49).

The distribution of food intake was assessed by calculating the percentage of TEI from six intervals throughout the day. Period 1 corresponded to the first moment of food consumption recorded until 8:59, period 2 was from 9:00 to 11:29, period 3 was from 11:30 to 14:29, period 4 was from 14:30 to 16:59, period 5 was from 17:00 to 19:59 and period 6 was from 20:00 until the time of the last food consumption recorded. The percentage of TEI from each period was calculated as the sum of the three-day energy intake from each period divided by the sum of the three-day TEI, multiplied by 100. These periods were based on hours delimiting periods in previous American and Canadian studies (6, 17, 18, 32) and adapted to the usual Canadian meal pattern (i.e., frequency and time of intakes) (50–52). They were also designed to capture late eating and main peaks of intake (i.e., meals) and snacks in different periods. Further details about the rationale behind the definition of time periods established in this study to assess the distribution of food intake have been previously published (53). To obtain the percentage of TEI from morning, afternoon and evening, the periods were combined as follows: morning (periods 1 and 2), afternoon (periods 3 and 4) and evening (periods 5 and 6). In the present study, we used the percentage of TEI consumed after 17:00 (i.e., periods 5 and 6 combined) and after 20:00 (period 6). A higher percentage of TEI consumed during the evening reflects a later distribution of food intake.

### 2.4. Eating behaviour traits and psychosocial factors

Eating behaviour traits were assessed with the Three-Factor Eating Questionnaire (TFEQ) (31, 54) and the Binge Eating Scale (BES) (55). The TFEQ is a 51-item questionnaire that measures three main dimensions of eating behaviour traits, namely cognitive restraint, disinhibition and susceptibility to hunger, with higher scores reflecting higher levels of these eating behaviours. Cognitive restraint reflects the tendency to restrain food intake to control or lose body weight. It is assessed with 21 items and can be separated into specific types, namely rigid and flexible restraint, each assessed with 7 items (56). Disinhibition is based on 16 items and reflects an overconsumption of food triggered by different cues representing its three subscales, namely habitual (5 items), emotional (3 items) and situational (5 items) susceptibility to disinhibition (57). Susceptibility to hunger (14 items) represents a susceptibility to experience feelings of hunger triggered by internal (internal locus of hunger, 6 items) or external (external locus of hunger, 6 items) cues (57). Thirty-six items are in a true or false format coded as 0 or 1 and 15 items are based on a 4 or 6-point scale coded as 0 or 1. The Binge Eating Scale assesses binge eating severity with 16 items describing the behavioural manifestations of binge eating and the feelings and cognitions surrounding binge eating episodes (55). Items are on a scale of 0 to 2 or 0 to 3, providing a total score of 0 to 46, with higher scores denoting higher binge eating severity.

Psychosocial factors, namely stress, anxiety and depressive symptoms were assessed with questionnaires. The Perceived Stress Scale measures the degree to which situations in one’s life are considered stressful during the last month (58). This questionnaire comprises 10 items assessed on a 5-point scale coded as 0 to 4, providing a total score ranging from 0 to 40 (59). The anxiety trait was assessed with 20 items of the State and Trait Anxiety Inventory (STAI) (60). These items are measured on a 4-point scale, ranging from 1 to 4, providing a total score for the trait section ranging between 20 and 80. Depressive symptoms were assessed with the Beck Depression Inventory (BDI) (61). The questionnaire comprises 21 items assessed on a 4 to 6-point scale, scored from 0 to 3, which provide a total score for the questionnaire ranging between 0 and 63. Higher scores on these questionnaires reflect higher levels of stress, anxiety or depressive symptoms (55, 58, 59, 61).

### 2.5. Assessment of covariates

Information on sex and age were collected at screening by the research staff. Sleep duration and habitual bedtime over the last month were self-reported with two questions based on the Pittsburgh Sleep Quality Index (62). Misreporting of energy intake was assessed by the method of Huang et al. (27) according to which under- and over reporting of energy intake are identified based on confidence limits around a ratio of self-reported energy intake (rEI) to total energy expenditure (TEE) calculated from a formula accounting for measurement error in rEI and TEE. Since an objective measure of resting metabolic rate (RMR) was available for 99% (*n* = 298) of participants, TEE was based on a factorial method using RMR and physical activity level (PAL) (63) rather than on equations such as those developed by the National Academy of Medicine as done in the original method of Huang et al. (27, 64). Resting metabolic rate was measured in a fasted state using indirect calorimetry in each study, as detailed elsewhere (65). For participants with missing data on RMR (*n* = 3), the latter was estimated using the Mifflin St-Jeor equation (66), which was found to be the most reliable equation to predict RMR in adults with normal weight and obesity, with the narrowest error range (67). Since participants had to be inactive to low active to be included in each study, RMR was multiplied by a PAL coefficient of 1.4 to determine TEE. A PAL coefficient of 1.4 represents the cut-off value between inactive and low active (64). Assuming a standard PAL for each participant in the assessment of misreporting of energy intake has been previously done (68). The ±1 standard deviation (SD) for confidence limits was established by using a within-individual coefficient of variation (CV) of 23.0% for rEI, n = 3 days for dietary assessment, a CV of 16.8% for TEE and a CV of 4.0% or 8.5% to account for within-individual day-to-day variation and error associated with objective measurement of TEE for measured or estimated RMR, respectively (69). To account for skewness of TEI, the ±1 SD confidence intervals were exponentiated using a multiplicative factor of 1 (70). For participants with an objective measure of RMR, the resulting confidence interval was 0.80 to 1.24 whereas the confidence interval was 0.79 to 1.26 for participants with estimated RMR. Individuals with values corresponding to or within the confidence interval were considered plausible reporters. Underreporting of energy intake was defined as rEI/TEE <0.80 and < 0.79 for participants with measured and estimated RMR, respectively. Overreporting of energy intake corresponded to rEI/TEE >1.24 and > 1.26 for participants with measured and estimated RMR, respectively. Based on these values, two indicator variables were created to represent underreporting (yes, 1; no, 0) and overreporting (yes, 1; no, 0). Because misreporting is based on a deviation from an exact correspondence between rEI and TEE (27), overreporting may also represent overconsumption in some individuals. Consequently, the main analyses were only adjusted for underreporting, but overreporting was further considered in supplemental analyses. Adjustment, rather than exclusion of under- and overreporters was used as it has been suggested as a more appropriate way to address misreporting since it avoids selection bias and reduction in statistical power (71). Ethnicity was also assessed at screening, but was not used as a covariate since there was low diversity in this sample.

### 2.6. Statistical analyses

Statistical analyses were performed using SAS version 9.4 (SAS Institute, Cary, NC). Sex differences in baseline characteristics, distribution of food intake, eating behaviour traits and psychosocial factors were assessed with Student’s T tests and Chi-square tests. Differences in these variables between studies were assessed with general linear models and Chi-square tests.

The associations between the percentage of TEI consumed in the evening (after 17:00 and after 20:00) and BMI, TEI, eating behaviour traits or psychosocial factors were assessed with Pearson correlations. The main analyses were adjusted for age, sex (men, 0; women, 1), underreporting of energy intake, sleep duration and bedtime. Due to sex differences in eating behaviour traits (34, 35), linear regressions were used to investigate sex interactions in these associations. In case of significant sex interaction, Pearson’s correlations were performed separately among men and women. The strength of associations for Pearson’s correlations was interpreted based on Funder and Ozer guidelines, where coefficients of 0.10, 0.20, and 0.30 represent small, moderate and large effect sizes, respectively (72).

Mediation analyses were conducted to assess if TEI mediates the potential associations between the percentage of TEI after 17:00 or after 20:00 and BMI. Mediation analyses were also performed to determine if the association between late eating and TEI is mediated by some eating behaviour traits that confer a susceptibility to overeating and showed a significant and positive association with late eating in correlation analyses. These analyses were performed separately among men and women when a sex interaction was previously observed in regression analyses. Mediation analyses were performed with model 4 of the Process macro for SAS, version 3.4.1 (73). Process is an ordinary least square regression path analysis modelling tool that assesses the mediating (or indirect) effect through which an independent variable influences a dependent variable using percentile bootstrap confidence intervals (73). The current study used 5,000 bootstrap samples. In the mediation model, the association between the independent variable and the mediator is represented by path *a*, and the association between the mediator and the dependent variable, adjusted for the independent variable, is represented by path *b*. The total effect (*c*) represents the association between the independent (late eating) and dependent variables (BMI or TEI) and the direct effect (*c’*) represents this same association but adjusted for the mediator (TEI or eating behaviour traits). Mediation analyses were adjusted for the same covariates as correlations. According to Hayes, a mediation effect could occur despite no evidence that the association between the independent and dependent variables is different from zero since the indirect effect is not determined nor constrained by the size of the total effect (73).

In secondary analyses, correlations and mediation models were adjusted for (1) age, sex and underreporting (i.e., model 2), (2) age, sex, misreporting (i.e., both under-and overreporting), sleep duration and bedtime (i.e., model 3) and (3) age, sex, underreporting, sleep duration, bedtime and studies (i.e., model 4). Adjustment for the different studies was performed by creating three indicator variables for Major et al. (42, 43) and Arguin et al. (41) studies. The Sanchez et al. (44) study was used as reference since this study had all of the questionnaires available and was performed in both men and women. For the associations between late eating and binge eating severity, perceived stress and anxiety, only one indicator variable for Sanchez et al. (44) was used as these questionnaires were only available in Arguin et al. (41) and Sanchez et al. (44).

## 3. Results

### 3.1. Participant characteristics

This study included 168 women and 133 men, with a mean age of 38.7 ± 8.5 years (range 19.7 to 55.2 years) and a mean BMI of 33.2 ± 3.4 kg/m^2^ (range 26.2 to 45.4 kg/m^2^) (Table 1). Participants consumed 43.7 ± 8.7% of TEI during the evening (after 17:00). Of this value, 9.2 ± 10.0% of TEI was consumed after 20:00. Except for TEI, there were no differences in these baseline characteristics nor distribution of food intake between men and women. However, as expected, there were several sex differences in eating behaviour traits, with women presenting higher levels of cognitive restraint (8.4 ± 4.0 vs. 5.8 ± 3.0, *p* < 0.0001) and emotional susceptibility to disinhibition (1.9 ± 1.2 vs. 1.4 ± 1.3, *p* = 0.002), and lower levels of situational susceptibility to disinhibition (3.3 ± 1.4 vs. 3.7 ± 1.2, *p* = 0.01) and susceptibility to hunger (5.7 ± 3.3 vs. 7.1 ± 3.6, *p* = 0.001). Women also reported higher levels of perceived stress (15.3 ± 6.0 vs. 12.5 ± 5.7, *p* = 0.002). Differences in participant characteristics, distribution of food intake, eating behaviour traits and psychosocial factors between studies included in this cohort are presented in Supplementary Table S1. Note that differences between studies are mainly attributed to sex.

**Table 1:** Participant characteristics.

	Total (*n* = 301)	Women (*n* = 168)	Men (*n* = 133)	*p*
Women, *n* (%)	168 (55.8)			0.04
Age, y	38.7 ± 8.5	38.0 ± 8.7	39.5 ± 8.1	0.14
Ethnicity, *n* (%)^a^				
White	284 (96.0)	161 (96.4)	123 (95.4)	0.65
Other	12 (4.1)	6 (3.6)	6 (4.7)	
BMI, kg/m^2^	33.2 ± 3.4	33.0 ± 3.5	33.5 ± 3.2	0.18
Total energy intake, kcal/day	2,524 ± 622	2,263 ± 541	2,853 ± 561	<0.0001
% Energy intake before 11:30	23.3 ± 7.2	21.1 ± 7.3	23.6 ± 7.1	0.50
% Energy intake between 11:30 and 16:59	33.0 ± 8.8	33.2 ± 8.6	32.7 ± 9.1	0.57
% Energy intake after 17:00	43.7 ± 8.7	43.7 ± 8.4	43.7 ± 9.0	0.98
% Energy intake after 20:00	9.2 ± 10.0	9.4 ± 9.7	9.0 ± 10.3	0.75
Reporting status, *n* (%)				
Underreporters	42 (14.0)	26 (15.5)	16 (12.0)	0.68
Plausible reporters	209 (69.4)	114 (67.9)	95 (71.4)	
Overreporters	50 (16.6)	28 (16.7)	22 (16.5)	
Eating behaviour traits^b^				
Cognitive restraint (0–21)	7.2 ± 3.8	8.4 ± 4.0	5.8 ± 3.0	<0.0001
Rigid restraint (0–7)	2.3 ± 1.6	2.7 ± 1.6	1.8 ± 1.4	<0.0001
Flexible restraint (0–7)	2.1 ± 1.6	2.5 ± 1.7	1.6 ± 1.3	<0.0001
Disinhibition (0–16)	8.5 ± 3.1	8.6 ± 3.0	8.4 ± 3.2	0.51
Habitual susceptibility (0–5)	1.6 ± 1.4	1.7 ± 1.4	1.4 ± 1.4	0.12
Emotional susceptibility (0–3)	1.7 ± 1.3	1.9 ± 1.2	1.4 ± 1.3	0.002
Situational susceptibility (0–5)	3.5 ± 1.3	3.3 ± 1.4	3.7 ± 1.2	0.01
Susceptibility to hunger (0–14)	6.3 ± 3.5	5.7 ± 3.3	7.1 ± 3.6	0.001
Internal locus of hunger (0–6)	2.4 ± 1.9	2.1 ± 1.8	2.7 ± 1.9	0.008
External locus of hunger (0–6)	2.8 ± 1.6	2.5 ± 1.6	3.1 ± 1.7	0.003
Binge eating severity (0–46)	12.9 ± 6.9	12.5 ± 6.4	13.2 ± 7.3	0.53
Psychosocial factors^c^				
Perceived stress (0–40)	13.6 ± 5.9	15.3 ± 6.0	12.5 ± 5.7	0.002
Anxiety -Trait (20–80)	36.6 ± 7.3	37.4 ± 7.0	36.1 ± 7.4	0.28
Depressive symptoms (0–63)	4.7 ± 4.9	5.1 ± 5.3	4.2 ± 4.2	0.11

a *n* = 296, Other: Black, *n* = 5, North African *n* = 3, Middle Eastern, *n* = 1, Hispanic, *n* = 2, mixed-race individuals, *n* = 1.

b Cognitive restraint, disinhibition and susceptibility to hunger, *n* = 261–289; Binge eating severity, *n* = 153.

c Perceived stress, *n* = 180, Anxiety trait, *n* = 172, depressive symptoms, *n* = 267. BMI, body mass index.

### 3.2. Associations between the percentage of TEI in the evening, TEI and BMI

There was a non-significant trend for the association between the percentage of TEI after 17:00 and BMI (*r* = 0.11, *p* = 0.07), but no association between the percentage of TEI after 20:00 and BMI (*r* = 0.05, *p* = 0.44). However, the percentage of TEI consumed after 17:00 or after 20:00 were positively associated with TEI (*r* = 0.13, *p* = 0.03 for both). No sex interaction was observed for these correlations (data not shown). Similar results were observed for correlations that did not consider sleep duration and bedtime or that were further adjusted for overreporting of energy intake or the different studies (Supplementary Table S2).

**Figure 1:**
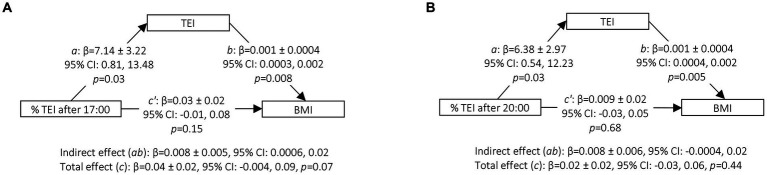
Mediation of TEI in the association between the percentage of TEI after 17:00 **(A)** or after 20:00 **(B)** and BMI. Mediation analyses were conducted using the Process Macro v. 3.4.1 for SAS that uses percentile bootstrap confidence intervals to assess the mediating or indirect effect. 95% CI for indirect effect are estimated through 5, 000 bootstrap samples. Models were adjusted for age (continuous), sex (men, 0; women, 1), underreporting of energy intake (yes, 1; no, 0), sleep duration (continuous) and bedtime (continuous). *a*, Association between the percentage of TEI after 17:00 or 20:00 and the TEI; *b*, Association between TEI and BMI adjusted for the percentage of TEI after 17:00 or after 20:00; total effect (*c*), association between the percentage of TEI after 17:00 or 20:00 and BMI without adjustment for the mediator (TEI); direct effect (*c’*), association between the percentage of TEI after 17:00 or 20:00 and BMI adjusted for the mediator (TEI); indirect effect (*ab*), mediation effect; Boot, Bootstrap; CI, confidence interval; TEI, total energy intake; BMI, body mass index. *n* = 278.

Mediation analyses showed a significant mediating effect of TEI on the association between the percentage of TEI after 17:00 and BMI (*β* = 0.008 ± 0.005, 95% CI: 0.0006, 0.02) (Figure 1A). Although of similar magnitude, the mediating effect for the model related to the percentage of TEI after 20:00 did not reach significance (*β* = 0.008 ± 0.006, 95% CI: −0.0004, 0.02) (Figure 1B). Similar results were observed after adjustment for the different sets of covariates (Supplementary Table S3). The model adjusted for age, sex and underreporting of energy intake showed a significant mediating effect of TEI in the association between the percentage of TEI after 20:00 and BMI, but the effect was similar to the original model.

### 3.3. Associations between the percentage of TEI in the evening, eating behaviour traits and psychosocial factors

The percentage of TEI after 17:00 was positively associated with disinhibition (*r* = 0.13, *p* = 0.03) (Table 2). The percentage of TEI after 20:00 was positively associated with habitual susceptibility to disinhibition (*r* = 0.13, *p* = 0.04), susceptibility to hunger (r = 0.13, *p* = 0.03), perceived stress (*r* = 0.24, *p* = 0.002) and anxiety trait (*r* = 0.28, *p* = 0.0004). A sex by distribution of food intake interaction indicated different patterns of association in men and women for some eating behaviour traits and depressive symptoms. The percentage of TEI after 17:00 was positively associated with disinhibition (*r* = 0.27, *p* = 0.0009) and its subscale habitual susceptibility to disinhibition (*r* = 0.21, *p* = 0.01) in women (Table 3). In men, the percentage of TEI after 17:00 was negatively associated with susceptibility to hunger (*r* = −0.20, *p* = 0.04) and its subscale external locus of hunger (*r* = −0.19, *p* = 0.04). Depressive symptoms were not associated with the percentage of TEI after 20:00 neither in men nor in women. Again, these results remained similar after adjustment for the different sets of covariates (Supplementary Tables S2, S4).

**Table 2:** Associations between the percentage of TEI after 17:00 or after 20:00, eating behaviour traits and psychosocial factors^a^.

	% TEI after 17:00			% TEI after 20:00		
	*r*	*p*	*p* sex int^b^	*r*	*p*	*p* sex int^b^
Eating behaviour traits^c^						
Cognitive restraint	0.05	0.47	0.48	0.03	0.67	0.44
Rigid restraint	0.09	0.14	0.90	−0.01	0.81	0.40
Flexible restraint	0.03	0.69	0.43	0.04	0.53	0.50
Disinhibition	**0.13**	**0.03**	**0.02**	0.09	0.13	0.61
Habitual susceptibility	0.10	0.12	**0.047**	**0.13**	**0.04**	0.84
Emotional susceptibility	0.09	0.15	0.28	0.10	0.11	0.23
Situational susceptibility	0.07	0.23	0.07	−0.01	0.87	0.89
Susceptibility to hunger	−0.04	0.47	**0.02**	**0.13**	**0.03**	0.54
Internal locus of hunger	−0.03	0.63	0.08	0.10	0.10	0.48
External locus of hunger	−0.06	0.35	**0.04**	0.07	0.23	0.82
Binge eating severity	0.05	0.56	0.64	0.12	0.19	0.65
Psychosocial factors^d^						
Perceived stress	0.12	0.13	0.51	**0.24**	**0.002**	0.65
Anxiety -Trait	0.10	0.22	0.85	**0.28**	**0.0004**	0.21
Depressive symptoms	0.05	0.40	0.50	0.02	0.75	**0.04**

a Values are Pearson correlation coefficients adjusted for age (continuous), sex (men, 0; women, 1), underreporting of energy intake (yes, 1; no, 0), sleep duration (continuous) and bedtime (continuous).

b *p* values for sex interaction obtained from linear regression. Int, interaction.

c Cognitive restraint, disinhibition and susceptibility to hunger, *n* = 249 to 276, Binge eating severity, *n* = 143.

d Psychosocial factors, *n* = 162 to 252. TEI, Total energy intake.

**Table 3:** Associations between the percentage of TEI after 17:00 or after 20:00 and eating behaviour traits or depressive symptoms, respectively, in men and women^a^.

	Women		Men	
	*r*	*p*	*r*	*p*
% of TEI after 17:00				
Disinhibition	**0.27**	**0.0009**	−0.03	0.76
Habitual susceptibility	**0.21**	**0.01**	−0.05	0.63
Susceptibility to hunger	0.09	0.25	**−0.20**	**0.04**
External locus of hunger	0.08	0.33	**−0.19**	**0.04**
% of TEI after 20:00				
Depressive symptoms	−0.10	0.23	0.17	0.08

^a^Values are Pearson correlation coefficients adjusted for age (continuous), underreporting (yes, 1; no, 0), sleep duration (continuous) and bedtime (continuous). Women, *n* = 146 to 154, men, *n* = 106 to 115. TEI, Total energy intake.

**Figure 2:**
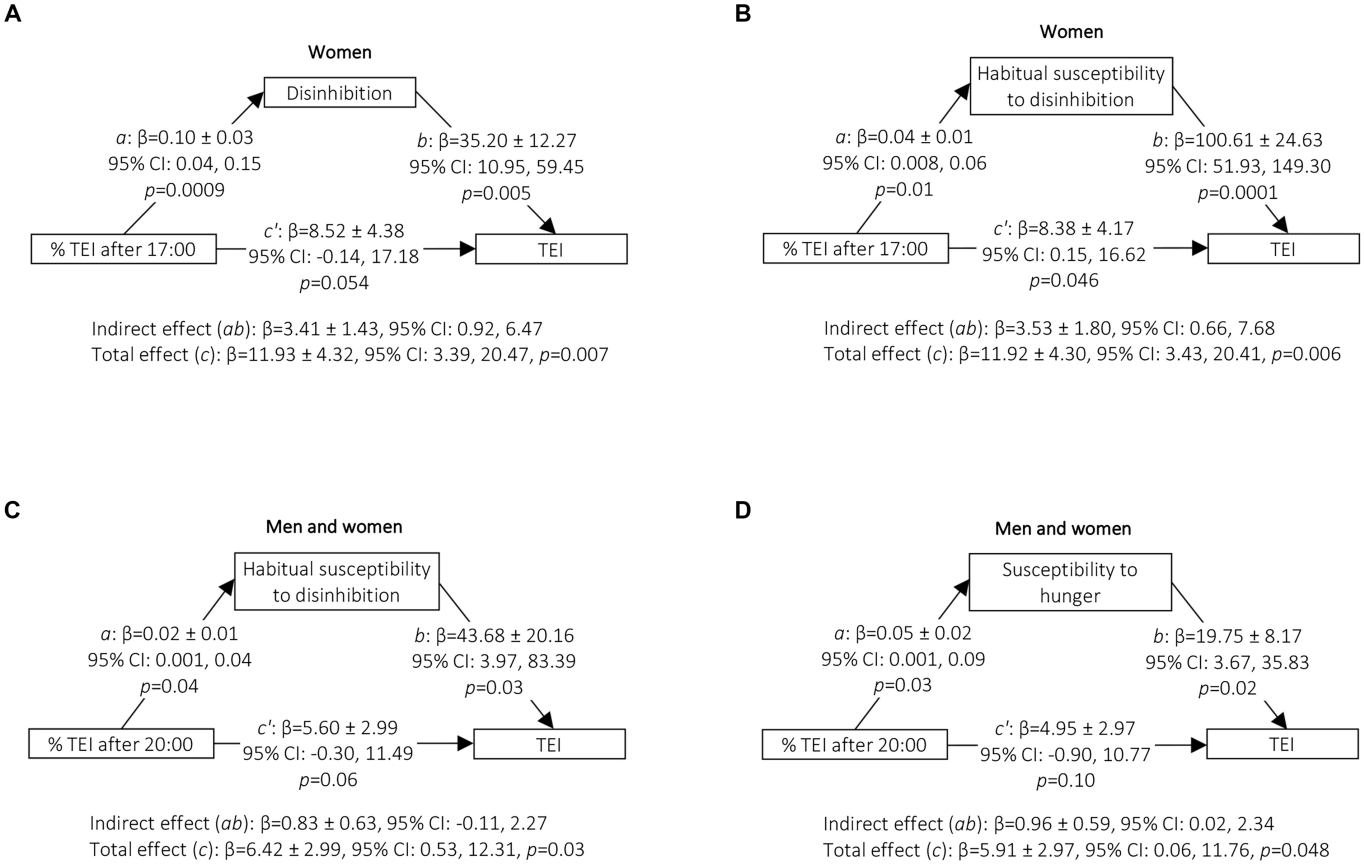
Mediation of disinhibition, habitual susceptibility to disinhibition and susceptibility to hunger in the association between the percentage of TEI after 17:00 or 20:00 and TEI in women (% TEI after 17:00) or in men and women (% TEI after 20:00). Mediation analyses were conducted using the Process Macro v. 3.4.1 for SAS that uses percentile bootstrap confidence intervals to assess the mediating or indirect effect. 95% CI for indirect effect are estimated through 5,000 bootstrap samples. Models were adjusted for age (continuous), underreporting of energy intake (yes, 1; no, 0), sleep duration (continuous) and bedtime (continuous). **(C,D)** are further adjusted for sex (men, 0; women, 1). *a*, association between percentage of TEI after 17:00 or after 20:00 and eating behaviour trait; *b*, association between eating behaviour trait and TEI adjusted for the percentage of TEI after 17:00 or after 20:00; total effect (*c*), association between the percentage of TEI after 17:00 or after 20:00 and TEI without adjustment for the mediator (eating behaviour trait); direct effect (*c’*), association between the percentage of TEI after 17:00 or after 20:00 and TEI adjusted for the mediator (eating behaviour trait); indirect effect (*ab*), mediation effect; Boot, Bootstrap; CI, confidence interval; TEI, total energy intake. **(A)** *n* = 152, **(B)** *n* = 147, **(C)** *n* = 259, **(D)** *n* = 267.

In women, mediation analyses showed that disinhibition and its subscale habitual susceptibility to disinhibition mediated the association between the percentage of TEI after 17:00 and TEI (*β* = 3.41 ± 1.43, 95% CI: 0.92, 6.47 and *β* = 3.53 ± 1.80, 95% CI: 0.66, 7.68, respectively) (Figures 2A,B). In men and women combined, susceptibility to hunger mediated the association between the percentage of TEI after 20:00 and TEI (*β* = 0.96 ± 0.59, 95% CI: 0.02, 2.34) (Figure 2D) but no mediating effect of habitual susceptibility to disinhibition in the association between the percentage of TEI after 20:00 and TEI was observed (*β* = 0.83 ± 0.63, 95% CI: −0.11, 2.27) (Figure 2C). These results remained similar after adjustment for the different sets of covariates (Supplementary Table S5).

## 4. Discussion

The timing of food intake has been identified as a risk factor for obesity. However, whether a late distribution of food intake impacts body weight through increased energy intake remains controversial and the behavioural characterization of late eaters needs to be further investigated. This study first examined the associations between a late distribution of food intake and TEI, BMI and eating and psychosocial traits, while considering underreporting of energy intake. This study also aimed to assess whether TEI and eating behaviour traits mediate the association between late eating and BMI or TEI, respectively. The results showed that a higher percentage of TEI during the evening was positively associated with TEI and that TEI mediated the association between the percentage of TEI after 17:00 and BMI. The results also showed that late eating was associated with higher levels of disinhibition, susceptibility to hunger, stress and anxiety, and that disinhibition in women and susceptibility to hunger in men and women combined mediated the association between late eating and TEI. Although cross-sectional, this study suggests that late eating could influence obesity through increased energy intake and that suboptimal eating behaviour traits could contribute to explaining the susceptibility to overeating among late eaters.

The association between the percentage of TEI in the evening and TEI was small, but consistent with previous observational studies (17–20). Moreover, although late eating (i.e., percentage of TEI after 17:00) showed a non-significant trend towards higher BMI, mediation analyses showed that late eating could be associated with BMI through higher energy intake. As indicated previously, an indirect effect can be different from zero even if the total effect is not (73). This is explained by the fact that the size of the indirect effect is not constrained nor determined by the size of the total effect (73). As shown in path *a* of Figures 1A,B each percent increase in TEI after 17:00 or after 20:00 resulted in an increase of approximately 7 kcal/day. When considering women only, the total effect (path *c*) of Figures 2A,B indicates that each percent increase in TEI after 17:00 results in an increase in energy intake of approximately 12 kcal/day. Through energy intake, the mediation models showed that each percent increase in TEI after 17:00 resulted in 0.008 unit of BMI. For an individual 1.75 m tall, an increase of 5% in TEI after 17:00 would result in an extra 0.12 kg of body weight. Given that the average increase in body weight among Canadian adults is 0.5 to 1 kg per 2-year period (74), the effect of the timing of food intake through energy intake may be a meaningful factor to consider in the aetiology of obesity.

Several factors may explain why a late distribution of food intake may lead to higher energy intake. Ghrelin, hunger, appetite for specific foods (i.e., sweets, salty and starchy foods, fruits and meat), desire to eat, prospective food consumption and fullness showed endogenous circadian rhythms with higher levels in the evening and lower levels in the morning and the opposite for fullness (75, 76). This potentially facilitates a positive energy balance towards the end of the day. These results were corroborated in an experimental study comparing appetite and food reward in response to a test meal during morning or late afternoon (77). Appetite, liking and wanting for high-fat foods were higher and post-meal fullness was lower in the late meal condition (77). The distribution of food intake may also influence appetite, as late eating resulted in higher daily levels of ghrelin and hunger and lower levels of fullness during weight loss (12). Results of this latter study were recently corroborated in two cross-over randomised controlled trials in which all foods were provided to participants during weight loss or weight stable conditions (25, 26). These studies reported higher daily levels of hunger, prospective food consumption and desire to eat in the late eating condition (25, 26). Homeostatic and hedonic mechanisms may thus be implicated in the effect of late eating on TEI.

The possible effect of late eating on energy intake is also supported by positive, small to moderate associations between late eating and eating behaviour traits associated with overeating, namely disinhibition and susceptibility to hunger. Moreover, these eating behaviours mediated the associations between late eating and TEI in women or in the whole group, respectively. These results are in line with previous studies showing that late eating was associated with overeating at night and emotional eating (13) and that evening snacking was positively associated with disinhibition (32). More broadly, these results are also in line with studies on chronotype showing that evening type individuals, who also present a delayed food pattern (78, 79), are more likely to present higher levels of disinhibition, susceptibility to hunger and binge eating and lower levels of cognitive restraint (80, 81). While cross-sectional, these results collectively suggest that late eating could lead to overconsumption through suboptimal eating behaviour traits.

Disinhibition behaviours and overconsumption triggered by susceptibility to hunger may be more likely to occur during the evening as a result of homeostatic and hedonic mechanisms potentially facilitating overeating during the evening presented above. Overeating associated with these eating behaviours may also be more likely to occur during the evening as a result of lower self-regulation capacity and increased tiredness (82), eating because others are eating (83, 84), eating in front of television (83), expecting eating to be more rewarding (83), alcohol consumption (83, 85), and more opportunities to eat alone (83) or when at home compared to at work or at school. It may also be a consequence of reduced energy intake during the day. Tani et al. showed that a lower proportion of TEI in the morning and in the afternoon were associated with higher energy intake during the evening, and a higher proportion of TEI during the evening was associated with higher TEI (19). A reduced energy intake earlier in the day may also be a consequence of overeating during the evening. Indeed, late eaters showed reduced morning appetite (13) and a Mendelian randomisation study showed that evening chronotype was causally associated with breakfast skipping, the latter also being causally associated with obesity (86).

Sex differences were observed in the associations between late eating and eating behaviour traits. Women with disinhibition seem more susceptible to overeating after 17:00, while both men and women presenting higher levels of susceptibility to hunger, showed a higher percentage of TEI after 20:00. This may be related to gender differences in social norms regarding food intake and body weight, as eating light meals are perceived as more feminine and heavy meals as more masculine (87). Eating smaller meals during the day may promote overeating in women with disinhibition during the evening, when at home, as opposed to men who may not perceive such social pressure to restrain eating during the day. Sex differences in chronotype may also be involved, as men usually present a later chronotype (78).

The positive associations between the proportion of TEI consumed after 20:00 and psychosocial factors such as stress and anxiety are in line with a previous study showing that late eaters were more likely to eat when stressed (13). More broadly, this is also in line with studies indicating that individuals with evening chronotype were more likely to experience negative psychological symptoms including anxiety (88), emotional overeating and stress-related eating (79, 89, 90). Because affective states and cognitive functioning worsen later in the day, resulting in a decrease in self-regulation capacity (82), it may be hypothesised that individuals with higher levels of stress and anxiety are more susceptible to overeating during the evening or at night. This hypothesis is supported by a study showing a positive association between psychological stress and self-reported overeating at dinner in workers (91). More broadly, this is also in line with the literature showing a positive association between emotional overeating and evening snacking (32) and that evening and night eating are used to regulate negative emotions among individuals with night eating syndrome (92). Participants of this latter study also reported that evening and night eating resulted in calmness (92). Lastly, a positive association between stress and hunger was found to be predominant during late afternoon and evening (93), which may also explain the increased susceptibility to overeating during the evening in individuals with stress and anxiety.

This study has several strengths and limitations. One of the main strengths is the use of a three-day food record with the consideration of underreporting of energy intake, and overreporting in supplemental analyses, to mitigate the effect of systematic bias in self-reported dietary assessment (27). As day-to-day variation was observed in the timing of food intake, combining several days of food intake is more likely to reflect a more habitual daily distribution of food intake than a single day (78, 94). The distribution of food intake was based on meal and snack times reported on the food record, which is more precise than using meals and snacks without considering hours. However, this classification is limited by not being defined relative to endogenous circadian timing or sleep–wake cycle (3). Yet, the consideration of mean sleep duration and bedtime over the last month as covariates suggests that the associations observed are not simply reflecting a shifted sleep–wake cycle providing more opportunities to eat in the evening in individuals with later bedtime and shorter sleep duration. It should be noted that these sleep parameters could have been more accurately assessed using objective measurements (e.g., accelerometer) or if sleep duration had been derived from self-reported data on usual bedtime, wake time, and length of time to fall asleep (95). One main limitation is the cross-sectional design that precludes causation to be inferred. Moreover, the relatively low sample size resulted in lower capacity to detect statistically significant results for some analyses. In addition, since the WeLIS cohort includes individuals living with overweight and obesity who are interested in losing weight, the results of this study may not be generalizable to other populations. Replication of these results within larger longitudinal cohorts and with objective measurements of food intake are needed.

In conclusion, this study suggests that late eating is positively associated with TEI, which may be related to higher BMI, in the long term. This association is supported by positive associations between late eating and eating behaviour traits, which contributed to explaining the susceptibility to overeating among late eaters. These results suggest that the timing of food intake and eating behaviour traits are important determinants to consider in obesity treatment and prevention.

## Data availability statement

The datasets presented in this article are not readily available because it will be made available upon request pending approval from the authors as well as the funding agency. Requests to access the datasets should be directed to vicky.drapeau@fse.ulaval.ca.

## Ethics statement

The studies involving human participants were reviewed and approved by Research Ethics Board of Université Laval. The participants provided their written informed consent to participate in their respective study.

## Author contributions

VD and RJ designed research. RJ collected data related to the timing of food intake, analyzed data, and wrote the first draught of the manuscript. VD had primary responsibility for the final content. All authors contributed to the article and approved the submitted version.

## Funding

Data collection that allowed the present study was partly funded by a grant of the Ministère de l’Enseignement supérieur, de la Recherche, de la Science et de la Technologie du Québec. RJ is the recipient of PhD scholarships from the Fonds de recherche du Québec–Santé (FRQS) and Canadian Institutes of Health Research (CIHR, MFE-171302). AT is the holder of the Canada Research Chair in Environment and Energy Balance. MEM holds a Canada Research Chair (Tier 1) in Physical Activity and Juvenile Obesity (950-232353). The funding agencies were not involved in designing and conducting the study, analyzing and interpreting the data or preparing and reviewing the manuscript before submission.

## Conflict of interest

The authors declare that the research was conducted in the absence of any commercial or financial relationships that could be construed as a potential conflict of interest.

## Publisher’s note

All claims expressed in this article are solely those of the authors and do not necessarily represent those of their affiliated organizations, or those of the publisher, the editors and the reviewers. Any product that may be evaluated in this article, or claim that may be made by its manufacturer, is not guaranteed or endorsed by the publisher.

## Abbreviations

TEI: total energy intake
Percent TEI: percentage of total energy intake
TFEQ: Three-Factor Eating Questionnaire
rEI: self-reported energy intake
TEE: total energy expenditure
RMR: resting metabolic rate
PAL: physical activity level
