# Supplementary material for: Associations of timing of food intake with energy intake, eating behaviour traits and psychosocial factors in adults with overweight and obesity
Source: Front Nutr. 2023 May 30;10:1155971. doi: 10.3389/fnut.2023.1155971 (PMC10267979; doi:10.3389/fnut.2023.1155971)
Supplement: Supplementary file 1 [file Data_Sheet_1.docx]

**Supplementary data**

Associations of timing of food intake with energy intake, eating behaviour traits and psychosocial factors in adults with overweight and obesity

Jacob R et al.

Online Supplemental Material

**Figure S1.** Flowchart diagram of participants' selection

Participants potentially eligible from baseline data from four weight loss studies

n=305

Participants included in the study

n=301

Exclusions:

1. Night shift work n=1
2. Food record with 1 or 2 days completed n=3

**Table S1.** Participant characteristics according to studies included

|  | Study 1  Major et al. (2007)  (n=65) | Study 2  Major et al.  (2008)  (n=44) | Study 3 Sanchez et al. (2014)  (n=126) | Study 4  Arguin et al. (2017)  (n=66) | *p* |
| --- | --- | --- | --- | --- | --- |
| Women, n (%) | 65 (100) ^*†‡^ | 25 (56.8) ^¶^ | 78 (61.9) ^\|\|^ | 0 (0) | <0.0001 |
| Age, y | 42.6 ± 5.5 ^*†^ | 36.3 ± 7.4 ^¶^ | 36.1 ± 10.0 ^\|\|^ | 41.4 ± 5.7 | <0.0001 |
| Ethnicity, n (%) ^1^ |  |  |  |  |  |
| White | 65 (100) | 40 (93.0) | 120 (95.2) | 59 (95.2) | 0.27 |
| Other | 0 (0) | 3 (7.0) | 6 (4.8) | 3 (4.8) |  |
| BMI, kg/m^2^ | 31.9 ± 3.1 ^*†‡^ | 34.3 ± 4.2 | 33.3 ± 3.3 | 33.5 ± 3.0 | 0.002 |
| Total energy intake, kcal/day | 2285 ± 390 ^*‡^ | 2713 ± 669 ^§^ | 2428 ± 648 ^\|\|^ | 2817 ± 590 | <0.0001 |
| % Energy intake before 11:30 | 21.3 ± 7.6 ^†^ | 21.8 ± 7.2 | 24.7 ± 6.5 | 23.5 ± 7.5 | 0.008 |
| % Energy intake between 11:30 and 16:59 | 33.9 ± 9.4 | 32.4 ± 8.4 | 32.9 ± 8.5 | 32.6 ± 9.2 | 0.81 |
| % Energy intake after 17:00 | 44.8 ± 7.9 | 45.8 ± 8.9 | 42.3 ± 9.0 | 43.9 ± 8.5 | 0.08 |
| % Energy intake after 20:00 | 8.5 ± 9.1 | 10.9 ± 13.2 | 9.7 ± 10.4 | 7.9 ± 7.0 | 0.41 |
| Reporting status, n (%) |  |  |  |  |  |
| Underreporters | 3 (4.6) | 6 (13.6) | 24 (19.1) | 9 (13.6) | 0.17 |
| Plausible reporters | 50 (76.9) | 28 (63.6) | 83 (65.9) | 48 (72.7) |  |
| Overreporters | 12 (18.5) | 10 (22.7) | 19 (15.1) | 9 (13.6) |  |
| **Eating behavior traits** |  |  |  |  |  |
| Cognitive restraint (0-21) | 8.0 ± 3.9 ^‡^ | 6.5 ± 4.0 ^§^ | 8.3 ± 3.8 ^\|\|^ | 5.0 ± 2.2 | <0.0001 |
| Rigid restraint (0-7) | 2.6 ± 1.6 ^‡^ | 2.0 ± 1.8 | 2.7 ± 1.5 ^\|\|^ | 1.5 ± 1.1 | <0.0001 |
| Flexible restraint (0-7) | 2.4 ±1.6 ^‡^ | 1.9 ± 1.3 | 2.3 ± 1.8 ^\|\|^ | 1.4 ± 1.1 | 0.0008 |
| Disinhibition (0-16) | 8.7 ± 3.0 | 9.3 ± 3.2 | 7.9 ± 3.0 | 8.9 ± 3.3 | 0.03 |
| Habitual susceptibility (0-5) | 1.8 ± 1.4 | 1.7 ± 1.4 | 1.4 ± 1.4 | 1.6 ± 1.5 | 0.32 |
| Emotional susceptibility (0-3) | 2.1 ± 1.1^†^ | 1.9 ± 1.2 ^§^ | 1.4 ± 1.3 | 1.7 ± 1.2 | 0.001 |
| Situational susceptibility (0-5) | 3.1 ± 1.4 ^‡^ | 3.6 ± 1.3 | 3.4 ± 1.3 | 3.8 ± 1.2 | 0.03 |
| Susceptibility to hunger (0-14) | 5.6 ± 3.4 ^‡^ | 5.7 ± 3.6 | 6.2 ± 3.4 | 7.4 ± 3.5 | 0.02 |
| Internal locus of hunger (0-6) | 2.1 ± 1.9 | 2.2 ± 1.9 | 2.3 ± 1.8 | 3.0 ± 2.0 | 0.06 |
| External locus of hunger (0-6) | 2.5 ± 1.6 | 2.6 ± 1.7 | 2.8 ± 1.7 | 3.2 ± 1.6 | 0.10 |
| Binge eating severity (0-46) | - | - | 12.1 ± 6.5 | 14.3 ± 7.4 | 0.052 |
| **Psychosocial factors** |  |  |  |  |  |
| Perceived stress (0-40) | - | - | 14.2 ± 6.0 | 12.5 ± 5.7 | 0.07 |
| Anxiety - Trait (20-80) | - | - | 37.1 ± 7.2 | 35.7 ± 7.2 | 0.20 |
| Depressive symptoms (0-63) | 4.5 ± 5.0 | 6.5 ± 6.6 | 4.6 ± 4.1 | 4.1 ± 4.3 | 0.08 |

* studies 1 vs 2, *p*<0.05; ^†^ studies 1 vs 3, *p*<0.05; ^‡^ studies 1 vs 4, *p*<0.05; ^§^ studies 2 vs 3, *p*<0.05; ^¶^ studies 2 vs 4, *p*<0.05; ^||^ studies 3 vs 4, *p*<0.05.

^1^ n=296; Other: Black, n=5, North African, n=3, Middle Eastern n=1, Hispanic, n=2, mixed-race individuals, n=1.

**Table S2.** Associations between the percentage of TEI after 17:00 or after 20:00, BMI, TEI, eating behavior traits and psychosocial factors with further adjustment for covariates

|  | % of TEI after 17:00 | | | | | | | |  | % of TEI after 20:00 | | | | | | | | |
| --- | --- | --- | --- | --- | --- | --- | --- | --- | --- | --- | --- | --- | --- | --- | --- | --- | --- | --- |
|  | **Model 2**  Adjusted for age, sex and undererporting | |  | **Model 3**  Adjusted for age, sex, misreporting, sleep duration and bedtime | |  | **Model 4**  Adjusted for age, sex, underreporting, sleep duration, bedtime and studies | |  | **Model 2**  Adjusted age, sex, and underreporting | |  | **Model 3**  Adjusted for age, sex, misreporting, sleep duration and bedtime | |  | **Model 4**  Adjusted for age, sex, underreporting, sleep duration, bedtime and studies | | |
|  | r | *p* |  | r | *p* |  | r | *p* |  | r | *p* |  | r | *p* |  | r | *p* |  |
| BMI | 0.09 | 0.14 |  | **0.12** | **0.047** |  | 0.10 | 0.11 |  | 0.02 | 0.68 |  | 0.08 | 0.21 |  | 0.04 | 0.51 |  |
| Total energy intake | **0.14** | **0.02** |  | **0.13** | **0.04** |  | 0.12 | 0.054 |  | **0.19** | **0.001** |  | 0.04 | 0.56 |  | **0.12** | **0.04** |  |
| **Eating behavior traits** |  |  |  |  |  |  |  |  |  |  |  |  |  |  |  |  |  |  |
| Cognitive restraint | 0.06 | 0.34 |  | 0.05 | 0.44 |  | 0.07 | 0.27 |  | 0.01 | 0.88 |  | 0.04 | 0.57 |  | 0.03 | 0.60 |  |
| Rigid restraint | 0.10 | 0.11 |  | 0.09 | 0.13 |  | 0.12 | 0.06 |  | -0.02 | 0.76 |  | -0.01 | 0.88 |  | -0.01 | 0.85 |  |
| Flexible restraint | 0.02 | 0.71 |  | 0.02 | 0.70 |  | 0.04 | 0.54 |  | 0.01 | 0.89 |  | 0.04 | 0.55 |  | 0.05 | 0.48 |  |
| Disinhibition | **0.13** | **0.03** |  | **0.13** | **0.04** |  | 0.11 | 0.08 |  | 0.08 | 0.18 |  | 0.08 | 0.18 |  | 0.09 | 0.15 |  |
| Habitual susceptibility | 0.11 | 0.06 |  | 0.09 | 0.14 |  | 0.08 | 0.19 |  | 0.10 | 0.11 |  | 0.12 | 0.06 |  | **0.12** | **0.496** |  |
| Emotional susceptibility | 0.08 | 0.16 |  | 0.08 | 0.18 |  | 0.06 | 0.32 |  | 0.11 | 0.07 |  | 0.08 | 0.20 |  | 0.09 | 0.13 |  |
| Situational susceptibility | 0.07 | 0.26 |  | 0.07 | 0.23 |  | 0.07 | 0.23 |  | -0.01 | 0.87 |  | -0.01 | 0.89 |  | -0.01 | 0.83 |  |
| Susceptibility to hunger | -0.04 | 0.49 |  | -0.05 | 0.45 |  | -0.04 | 0.49 |  | **0.14** | **0.02** |  | **0.13** | **0.03** |  | **0.14** | **0.03** |  |
| Internal locus of hunger | -0.03 | 0.63 |  | -0.03 | 0.61 |  | -0.03 | 0.60 |  | 0.10 | 0.10 |  | 0.10 | 0.11 |  | 0.11 | 0.09 |  |
| External locus of hunger | -0.05 | 0.41 |  | -0.06 | 0.33 |  | -0.05 | 0.38 |  | 0.07 | 0.23 |  | 0.07 | 0.28 |  | 0.08 | 0.22 |  |
| Binge eating severity | 0.04 | 0.66 |  | 0.06 | 0.51 |  | 0.04 | 0.62 |  | 0.09 | 0.28 |  | 0.15 | 0.08 |  | 0.11 | 0.18 |  |
| **Psychosocial factors** |  |  |  |  |  |  |  |  |  |  |  |  |  |  |  |  |  |  |
| Perceived stress | 0.12 | 0.11 |  | 0.12 | 0.13 |  | 0.12 | 0.13 |  | **0.21** | **0.006** |  | **0.23** | **0.003** |  | **0.24** | **0.002** |  |
| Anxiety - Trait | 0.11 | 0.17 |  | 0.10 | 0.22 |  | 0.10 | 0.21 |  | **0.26** | **0.0008** |  | **0.28** | **0.0004** |  | **0.28** | **0.0004** |  |
| Depressive symptoms | 0.08 | 0.18 |  | 0.05 | 0.44 |  | 0.03 | 0.65 |  | 0.08 | 0.22 |  | 0.01 | 0.87 |  | 0.004 | 0.96 |  |

BMI and total energy intake, model 2: n=301, models 3 and 4: n=278; Cognitive restraint, disinhibition and susceptibility to hunger, model 2: n=261 to 289, models 3 and 4: n=249 to 276; Binge eating severity, model 2: n=153, model 3 and 4: n=143; Psychosocial factors, model 2: n=172 to 267, model 3 and 4: n=162 to 252. Values are partial Pearson correlation coefficients. Covariates: age (continuous), sex (men, 0, women, 1), underreporting of energy intake (yes, 1; no, 0), overreporting of energy intake (yes, 1; no, 0), sleep duration (continuous), bedtime (continuous), studies (study 1 [Major et al. 2007], yes, 1, no, 0; study 2 [Major et al. 2008], yes, 1, no, 0; and study 4 [Arguin et al. 2017], yes, 1, no, 0; or only study 3 [Sanchez et al. 2014], yes 1, no, 0 for binge eating severity, perceived stress and anxiety trait), depending on the models. TEI, total energy intake, BMI, body mass index. Bold values indicate significant correlations.

**Table S3.** Mediation of TEI in the association between the percentage of TEI after 17:00 or after 20:00 and BMI with further adjustments for covariates

|  | *a* | | |  | *b* | | |  | Total effect (*c*) | | |  | Direct effect | | |  | Indirect effect (*ab*) | | |
| --- | --- | --- | --- | --- | --- | --- | --- | --- | --- | --- | --- | --- | --- | --- | --- | --- | --- | --- | --- |
|  | β ± SE | 95% CI | *p* |  | β ± SE | 95% CI | *p* |  | β ± SE | 95% CI | *p* |  | β ± SE | 95% CI | *p* |  | β ± Boot SE | Boot 95% CI |  |
| **% TEI after 17:00 → TEI → BMI** |  |  |  |  |  |  |  |  |  |  |  |  |  |  |  |  |  |  |  |
| Model 2: Adjusted for age, sex and underreporting | 7.64 ± 3.14 | 1.47, 13.81 | 0.02 |  | 0.001 ± 0.0004 | 0.0003, 0.002 | 0.008 |  | 0.03 ± 0.02 | -0.01, 0.08 | 0.14 |  | 0.03 ± 0.02 | -0.02, 0.07 | 0.26 |  | **0.008 ± 0.005** | **0.0008, 0.02** |  |
| Model 3: Adjusted for age, sex, misreporting, sleep duration and bedtime | 5.25 ± 2.52 | 0.28, 10.21 | 0.04 |  | 0.003 ± 0.0005 | 0.002, 0.004 | <0.0001 |  | 0.05 ± 0.02 | 0.0006, 0.09 | 0.047 |  | 0.03 ± 0.02 | -0.01, 0.07 | 0.18 |  | **0.02 ± 0.009** | **0.0005, 0.04** |  |
| Model 4: Adjusted for age, sex, underreporting, sleep duration, bedtime and studies | 6.27 ± 3.25 | -0.12, 12.66 | 0.054 |  | 0.001 ± 0.0004 | 0.0002, 0.002 | 0.01 |  | 0.04 ± 0.02 | -0.008, 0.08 | 0.11 |  | 0.03 ± 0.02 | -0.01, 0.08 | 0.18 |  | **0.007 ± 0.005** | **0.0001, 0.02** |  |
| **% TEI after 20:00 → TEI → BMI** |  |  |  |  |  |  |  |  |  |  |  |  |  |  |  |  |  |  |  |
| Model 2: Adjusted for age, sex and underreporting | 9.04 ± 2.73 | 3.66, 14.42 | 0.001 |  | 0.001 ± 0.0004 | 0.0003, 0.002 | 0.005 |  | 0.008 ± 0.02 | -0.03, 0.05 | 0.68 |  | -0.002 ± 0.02 | -0.04, 0.04 | 0.90 |  | **0.01 ± 0.006** | **0.001, 0.02** |  |
| Model 3: Adjusted for age, sex, misreporting, sleep duration and bedtime | 1.40 ± 2.37 | -3.27, 6.06 | 0.56 |  | 0.003 ± 0.0005 | 0.002, 0.004 | <0.0001 |  | 0.03 ± 0.02 | -0.02, 0.07 | 0.21 |  | 0.02 ± 0.02 | -0.02, 0.06 | 0.26 |  | 0.005 ± 0.009 | -0.01, 0.02 |  |
| Model 4: Adjusted for age, sex, underreporting, sleep duration, bedtime and studies | 6.01 ± 2.96 | 0.18, 11.83 | 0.04 |  | 0.001 ± 0.0004 | 0.0003, 0.002 | 0.01 |  | 0.01 ± 0.02 | -0.03, 0.06 | 0.51 |  | 0.007 ± 0.02 | -0.03, 0.05 | 0.73 |  | 0.007 ± 0.005 | -0.0007, 0.02 |  |

Model 2: n=301, Models 3 and 4: n=278. Mediation analyses are conducted using the Process Macro v. 3.4.1 for SAS that uses percentile bootstrap confidence intervals to assess the mediating or indirect effect. 95% CI for indirect effect are estimated through 5, 000 bootstrap samples. Covariates: age (continuous), sex (men, 0; women, 1), underreporting of energy intake (yes, 1; no, 0), overreporting of energy intake (yes, 1; no, 0), sleep duration (continuous), bedtime (continuous), studies (study 1 [Major et al. 2007], yes, 1; no, 0; study 2 [Major et al. 2008], yes, 1; no, 0; and study 4 [Arguin et al. 2017], yes, 1; no, 0), depending on the models. *a*, association between % EI after 17:00 or 20:00 and TEI; *b*, association between TEI and BMI adjusted for % EI after 17:00 or after 20:00; total effect (*c*), association between % EI after 17:00 or 20:00 and BMI without adjustment for the mediator (TEI); direct effect (*c'*), association between % EI after 17:00 or 20:00 and BMI adjusted for the mediator (TEI); indirect effect (*ab*), mediation effect; Boot, Bootstrap; CI, confidence interval; EI, energy intake; TEI, total energy intake; BMI, body mass index. Bold values indicates significant indirect effect (mediation).

**Table S4.** Associations among the percentage of TEI after 17:00 of after 20:00 and eating behavior traits or depressive symptoms in men and, with further adjustment for covariates

|  | Women | | | | | | | |  | Men | | | | | | | | |
| --- | --- | --- | --- | --- | --- | --- | --- | --- | --- | --- | --- | --- | --- | --- | --- | --- | --- | --- |
|  | **Model 2**  Adjusted for age and undererporting | |  | **Model 3**  Adjusted for age, misreporting, sleep duration and bedtime | |  | **Model 4**  Adjusted for age, underreporting, sleep duration, bedtime and studies | |  | **Model 2**  Adjusted age and underreporting | |  | **Model 3**  Adjusted for age, misreporting, sleep duration and bedtime | |  | **Model 4**  Adjusted for age, underreporting, sleep duration, bedtime and studies | | |
|  | r | *p* |  | r | *p* |  | r | *p* |  | r | *p* |  | r | *p* |  | r | *p* |  |
| **% TEI after 17:00** |  |  |  |  |  |  |  |  |  |  |  |  |  |  |  |  |  |  |
| Disinhibition | **0.25** | **0.002** |  | **0.26** | **0.002** |  | **0.24** | **0.004** |  | -0.001 | 0.99 |  | -0.03 | 0.76 |  | -0.04 | 0.69 |  |
| Habitual susceptibility | **0.22** | **0.008** |  | **0.18** | **0.03** |  | **0.18** | **0.03** |  | -0.01 | 0.93 |  | -0.05 | 0.62 |  | -0.05 | 0.61 |  |
| Susceptibility to hunger | 0.09 | 0.26 |  | 0.08 | 0.33 |  | 0.10 | 0.22 |  | **-0.19** | **0.03** |  | **-0.20** | **0.04** |  | **-0.20** | **0.04** |  |
| External locus of hunger | 0.08 | 0.31 |  | 0.06 | 0.45 |  | 0.09 | 0.30 |  | **-0.19** | **0.03** |  | **-0.19** | **0.04** |  | **-0.19** | **0.045** |  |
| **% TEI after 20:00** |  |  |  |  |  |  |  |  |  |  |  |  |  |  |  |  |  |  |
| Depressive symptoms | 0.01 | 0.87 |  | -0.12 | 0.14 |  | -0.12 | 0.17 |  | 0.16 | 0.09 |  | 0.17 | 0.08 |  | 0.16 | 0.11 |  |

Women: model 2, n=152 to 160, models 3 and 4, n=146 to 152, Men: model 2, n=114 to 123, models 3 and 4, n=106 to 115. Values are partial Pearson correlation coefficients. Covariates: age (continuous), underreporting of energy intake (yes, 1; no, 0), overreporting of energy intake (yes, 1; no, 0), sleep duration (continuous), bedtime (continuous), studies (study 1 [Major et al. 2007], yes, 1; no, 0; study 2 [Major et al. 2008], yes, 1; no, 0; and study 4 [Arguin et al. 2017], yes, 1; no, 0), depending on the models. TEI, total energy intake. Bold values indicate significant correlations.

**Table S5.** Mediation of disinhibition, habitual susceptibility to disinhibition and susceptibility to hunger in the association between the percentage of TEI after 17:00 or 20:00 and TEI in women (% TEI after 17:00) or in men and women (% TEI after 20:00) with further adjustment for covariates.

|  | *a* | | |  | *b* | | |  | Total effect (*c*) | | |  | Direct effect | | |  | Indirect effect (*ab*) | |
| --- | --- | --- | --- | --- | --- | --- | --- | --- | --- | --- | --- | --- | --- | --- | --- | --- | --- | --- |
|  | β ± SE | 95% CI | *p* |  | β ± SE | 95% CI | *p* |  | β ± SE | 95% CI | *p* |  | β ± SE | 95% CI | *p* |  | β ± Boot SE | Boot 95% CI |
| **% TEI after 17:00 → Disinhibition → TEI (in women)** |  |  |  |  |  |  |  |  |  |  |  |  |  |  |  |  |  |  |
| Model 2: Adjusted for age and underreporting | 0.09 ± 0.03 | 0.03, 0.14 | 0.002 |  | 41.31 ± 12.19 | 17.23, 65.39 | 0.0009 |  | 12.26 ± 4.28 | 3.81, 20.72 | 0.005 |  | 8.70 ± 4.27 | 0.26, 17.13 | 0.04 |  | **3.57 ± 1.45** | **0.96, 6.68** |
| Model 3: Adjusted for age, misreporting, sleep duration and bedtime | 0.09 ± 0.03 | 0.03, 0.15 | 0.002 |  | 25.48 ± 9.98 | 5.76 , 45.21 | 0.01 |  | 7.53 ± 3.51 | 0.59, 14.46 | 0.03 |  | 5.19 ± 3.56 | -1.85, 12.23 | 0.15 |  | **2.33 ± 1.09** | **0.46, 4.71** |
| Model 4: Adjusted for age, underreporting, sleep duration, bedtime and studies | 0.09 ± 0.03 | 0.03, 0.14 | 0.004 |  | 33.45 ± 12.44 | 8.85, 58.04 | 0.008 |  | 10.69 ± 4.42 | 1.95, 19.43 | 0.02 |  | 7.81 ± 4.46 | -1.00, 16.63 | 0.08 |  | **2.87 ± 1.33** | **0.60, 5.78** |
| **% TEI after 17:00 → Habitual susceptibility to disinhibition →TEI (in women)** |  |  |  |  |  |  |  |  |  |  |  |  |  |  |  |  |  |  |
| Model 2: Adjusted for age and underreporting | 0.04 ± 0.01 | 0.01, 0.06 | 0.008 |  | 110.26 ± 24.85 | 61.14, 159.38 | <0.0001 |  | 12.64 ± 4.27 | 4.20, 21.09 | 0.004 |  | 8.69 ± 4.12 | 0.54, 16.84 | 0.04 |  | **3.96 ± 1.79** | **0.73, 7.61** |
| Model 3: Adjusted for age, misreporting, sleep duration and bedtime | 0.03 ± 0.01 | 0.003, 0.06 | 0.03 |  | 69.68 ± 20.68 | 28.78, 110.58 | 0.001 |  | 7.53 ± 3.52 | 0.57, 14.50 | 0.03 |  | 5.39 ± 3.46 | -1.44, 12.23 | 0.12 |  | **2.14 ± 1.19** | **0.10, 5.50** |
| Model 4: Adjusted for age, underreporting, sleep duration, bedtime and studies | 0.03 ± 0.01 | 0.003, 0.06 | 0.03 |  | 98.30 ± 24.89 | 49.08, 147.51 | 0.0001 |  | 10.64 ± 4.40 | 1.95, 19.33 | 0.02 |  | 7.59 ± 4.25 | -0.82, 15.99 | 0.08 |  | **3.05 ± 1.77** | **0.24, 7.12** |
| **% TEI after 20:00 → Habitual susceptibility to disinhibition →TEI (in men and women)** |  |  |  |  |  |  |  |  |  |  |  |  |  |  |  |  |  |  |
| Model 2: Adjusted for age, sex and underreporting | 0.01 ± 0.009 | -0.003, 0.03 | 0.11 |  | 42.32 ± 19.49 | 3.95, 80.69 | 0.03 |  | 9.13 ± 2.78 | 3.66, 14.59 | 0.001 |  | 8.54 ± 2.77 | 3.08, 13.99 | 0.002 |  | 0.59 ± 0.53 | -0.23, 1.84 |
| Model 3: Adjusted for age, sex, misreporting, sleep duration and bedtime | 0.02 ± 0.009 | -0.001, 0.04 | 0.06 |  | 34.41 ± 16..42 | 2.07, 66.75 | 0.04 |  | 1.99 ± 2.46 | -2.86, 8.84 | 0.42 |  | 1.38 ± 2.46 | -3.47, 6.23 | 0.58 |  | 0.61 ± 0.51 | -0.13, 1.83 |
| Model 4: Adjusted for age, sex, underreporting, sleep duration, bedtime and studies | 0.02 ± 0.009 | 0.00, 0.04 | 0.0496 |  | 41.92 ± 20.23 | 2.07, 81.77 | 0.04 |  | 6.23 ± 2.98 | 0.36, 12.10 | 0.04 |  | 5.46 ± 2.98 | -0.41, 11.34 | 0.068 |  | 0.77 ± 0.61 | -0.14, 2.20 |
| **% TEI after 20:00 → susceptibility to hunger → TEI (in men and women)** |  |  |  |  |  |  |  |  |  |  |  |  |  |  |  |  |  |  |
| Model 2: Adjusted for age, sex and underreporting | 0.05 ± 0.02 | 0.009, 0.09 | 0.02 |  | 24.32 ± 7.99 | 8.59, 40.04 | 0.003 |  | 8.65 ± 2.74 | 3.25, 14.05 | 0.002 |  | 7.45 ± 2.73 | 2.07, 12.83 | 0.007 |  | **1.20 ± 0.67** | **0.13, 2.73** |
| Model 3: Adjusted for age, sex, misreporting, sleep duration and bedtime | 0.05 ± 0.02 | 0.004, 0.09 | 0.03 |  | 19.39 ± 6.48 | 6.64, 32.15 | 0.003 |  | 1.18 ± 2.40 | -3.55, 5.91 | 0.62 |  | 0.24 ± 2.39 | -4.46, 4.94 | 0.92 |  | **0.94 ± 0.54** | **0.07, 2.16** |
| Model 4: Adjusted for age, sex, underreporting, sleep duration, bedtime and studies | 0.05 ± 0.02 | 0.005, 0.09 | 0.03 |  | 21.35 ± 8.17 | 5.25, 37.48 | 0.01 |  | 5.63 ± 2.97 | -0.21, 11.48 | 0.06 |  | 4.57 ± 2.96 | -1.26, 10.41 | 0.12 |  | **1.06 ± 0.60** | **0.07, 2.43** |

% TEI after 17:00 → Disinhibition → TEI (in women), n=152 to 158; % TEI after 17:00 → Habitual susceptibility to disinhibition →TEI (in women), n=147 to 152; % TEI after 20:00 → Habitual susceptibility to disinhibition →TEI (in men and women), n=259 to 272; % TEI after 20:00 → susceptibility to hunger → TEI (in men and women), n=267 to 281. Mediation analyses are conducted using the Process Macro v. 3.4.1 for SAS that uses percentile bootstrap confidence intervals to assess the mediating or indirect effect. 95% CI for indirect effect are estimated through 5, 000 bootstrap samples. Covariates: age (continuous), sex (men, 0; women, 1), underreporting of energy intake (yes, 1; no, 0), overreporting of energy intake (yes, 1; no, 0)], sleep duration (continuous), bedtime (continuous), studies (study 1 [Major et al. 2007], yes, 1; no, 0; study 2 [Major et al. 2008], yes, 1; no, 0; and study 4 [Arguin et al. 2017], yes, 1; no, 0), depending on the models. *a*, association between % TEI after 17:00 or 20:00 and eating behavior traits; *b*, association between eating behavior trait and TEI adjusted for % TEI after 17:00 or after 20:00; total effect (*c*), association between % TEI after 17:00 or 20:00 and TEI without adjustment for the mediator (eating behavior trait); direct effect (*c'*), association between % TEI after 17:00 or 20:00 and TEI adjusted for the mediator (eating behavior trait); indirect effect (*ab*), mediation effect; Boot, Bootstrap; CI, confidence interval; TEI, total energy intake; BMI, body mass index. Bold values indicate significant indirect effect (mediation).
